# Long-Term Prognostic Validity of Talent Selections: Comparing National and Regional Coaches, Laypersons and Novices

**DOI:** 10.3389/fpsyg.2017.01146

**Published:** 2017-07-11

**Authors:** Jörg Schorer, Rebecca Rienhoff, Lennart Fischer, Joseph Baker

**Affiliations:** ^1^Institute of Sport Science, University of Oldenburg Oldenburg, Germany; ^2^Institute of Sport and Exercise Sciences, University of Münster Münster, Germany; ^3^School of Kinesiology and Health Science, York University, Toronto ON, Canada

**Keywords:** talent selection, decision making, handball, motor tests, longitudinal design, coaches

## Abstract

In most sports, the development of elite athletes is a long-term process of talent identification and support. Typically, talent selection systems administer a multi-faceted strategy including national coach observations and varying physical and psychological tests when deciding who is chosen for talent development. The aim of this exploratory study was to evaluate the prognostic validity of talent selections by varying groups 10 years after they had been conducted. This study used a unique, multi-phased approach. Phase 1 involved players (*n* = 68) in 2001 completing a battery of general and sport-specific tests of handball ‘talent’ and performance. In Phase 2, national and regional coaches (*n* = 7) in 2001 who attended training camps identified the most talented players. In Phase 3, current novice and advanced handball players (*n* = 12 in each group) selected the most talented from short videos of matches played during the talent camp. Analyses compared predictions among all groups with a best model-fit derived from the motor tests. Results revealed little difference between regional and national coaches in the prediction of future performance and little difference in forecasting performance between novices and players. The best model-fit regression by the motor-tests outperformed all predictions. While several limitations are discussed, this study is a useful starting point for future investigations considering athlete selection decisions in talent identification in sport.

## Introduction

One of the most important and difficult tasks in our society is forecasting a person’s potential. In music, dance, and theater, choices regarding who is cast are made based on a few minutes interaction with potential candidates. Similarly, top-rated reality TV-shows like American Idol or Australia’s Next Top Model attempt to forecast potential based on sometimes very brief performance exposures. In each of these situations decisions are made on the basis of available information and assumptions about how this information relates to future performance. In sport, the entire field of talent identification is grounded in the notion that performance during early stages of development gives some indicator of an athlete’s future potential (Baker et al., 2017).

One of the cornerstones of sport science is understanding the factors explaining exceptional performance (Starkes and Ericsson, 2003; Baker and Farrow, 2015). Although this information is valuable for appreciating the physical, cognitive and psychological capabilities of our species as well as for testing models of how humans respond under intense stress, its primary *raison d’être* is usually to provide coaches and trainers with information to improve judgments about talent selection and development. Despite its potential importance to sports systems worldwide, there has been very little research considering whether performance parameters measured earlier in development (e.g., in youth) are valid predictors of expertise later in development (e.g., as an adult).

One of the factors limiting research in this area is the difficulty of obtaining high quality youth samples and managing to track them over extended periods of time in order to determine the level of performance they ultimately attain. Moreover, because most talent development systems operate via a ‘de-selection’ process (i.e., most athletes are removed from the system with a select minority being chosen for continued advancement) the number of athletes who start in the system and the number exiting the system at its highest level are considerably different, which increases the administrative, logistical and financial demands on researchers aiming to obtain samples of sufficient size for inferential and comparative analyses. Although demanding, longitudinal studies of talent development have begun to emerge – most notably from Elferink-Gemser, Visscher and their colleagues as well as Till et al. (2013, 2015) and Deprez et al. (2014, 2015a,b,c). These studies have been extremely useful in identifying the varying influence of a range of variables, such as self-regulation, quantity of practice, and early measures of technical and tactical skill on the long-term development of athletes in soccer, field hockey and rugby, among other sports. Within sport science almost all talent prediction research is based on the assumption that there is a ‘formula’ that could identify athletes best suited for specific sports. An extreme example for this was the talent system in the former German Democratic Republic, in which all school children were scanned with various tests to look for students with talent (Kupper and Thieß, 1971). The assumed linearity underpinning these approaches (e.g., someone who performs well at the talent selection stage will perform exceptionally in later years) has been criticized from an ecological perspective (Renshaw et al., 2012), resulting in these approaches having had negligible effects on improving the efficacy of talent selection decisions.

Available evidence of the accuracy of talent decisions by coaches and scouts is not compelling. Koz et al.’s (2012) study of the accuracy of selection decisions for professional sports ‘entry drafts’ suggests that even when these decisions are made late in development (i.e., early adulthood), the level of predictive accuracy is relatively low. However, these types of archival analyses (see also Staw and Hoang, 1995; Berri and Simmons, 2009) may provide negligible information regarding the quality and accuracy of decision-making during early development (e.g., during youth). While there is some suggestion that coaches experience and training may allow them to see something less qualified individuals have difficulty seeing (Christensen, 2009), this notion has not been adequately examined using comparison-based designs.

In the sections below we describe the results of an analysis that evolved over an extended timespan. One of the authors was involved in the initial assessment of a group of youth handball players over 10 years ago and we are now able to determine how these athletes advanced across their athletic careers. The primary aim of this exploratory study was to evaluate the prognostic validity of talent selections by varying groups 10 years after they had been conducted. In a first step of this study, motor test results acquired during a youth talent camp were used to determine a possible model fit. Following Schneider et al. (1993), these tests are often used to measure general motor abilities of young athletes (e.g., Söğüt, 2016) and are widely administered by many sports for talent selection (e.g., Schorer et al., 2012). While often administered, the use of motor tests seems debatable (Lidor et al., 2009), although these tests have had some success in handball (Lidor et al., 2005; Matthys et al., 2013). While Matthys et al. (2013) found some differences between elite and non-elite players in the Yo-Yo intermittent recovery test and on the speed and coordination items, in the study by Lidor et al. (2005) only a handball skill specific test differentiated.

In part two of this study, the original subjective predictions of national and regional coaches who scouted during the tournament of this youth talent camp were evaluated. Determining the long-term accuracy of their predictions is the main focus of our study. In part three of this study, these coach predictions were compared to those made by amateur handball players and novices (i.e., no handball experience) who viewed short videos from the tournament matches. This final element was included to compare the expert decisions of the national and regional coaches with advanced performers (i.e., players) and novices (Abernethy et al., 1993) to determine whether the accuracy of prediction was differentiated by skill level. Because this comparison was conducted after the talent selection occurred, the amount of information given to these groups was limited. Therefore, these groups serve only as a baseline comparison to the performance of the national and regional coaches.

Because much of the research design was determined by serendipity as much as it was by conscious planning, we are cautious in how we present these results; however, given the lack of data on the efficacy of coach/scout decision-making in talent settings, we believe the comparisons (i.e., the motor tests, coach decisions, and players and novice decisions) provide us with the opportunity to explore the prognostic validity of talent selections over an extended period (cf. Vaeyens et al., 2009; Rees et al., 2016).

## Materials and Methods

This study was conducted in three parts. These parts evolved over time and are presented in temporal order below.

### Part 1: Prediction by Motor Test

#### Participants

Sixty-eight female handball players participated in a talent camp, 56 born in the older year (age 13–14) and 12 born in the younger year (age 12–13). All participants were chosen by regional coaches to participate in the talent camp as a preparation for the national talent selection. Twelve of the 68 players in this camp were invited to the national talent selection of the German Handball Federation. Ten years later, 16 of the original 68 players were playing handball as professionals, four in the first German Handball league and twelve in the second league. Two were later National team members. These 16 were considered as ‘high achievers.’ The remaining 50 players either were not playing handball any longer or were playing in lower leagues. These were considered as ‘low achievers.’

#### Procedures

The 2001 talent selection camp used a range of motor tests to identify the best players of the 68 participants. These tests collected anthropometric data such as body height and body weight, performance data from handball specific games (e.g., 4 vs. 4 or 3 vs. 3 players) as well as data from 12 basic fitness and coordinative tasks. These 12 tasks are described below.

##### Hover stand

In this task, participants had to stand with one foot on a wooden block (2 cm wide and 10 cm high) and balance for as long as possible. Hands are kept on the hip and the “free foot” can be used to assist balance. Time was measured until either one foot touched the floor, the hands moved away from the hip or 1 min elapsed. The best of two trials was recorded.

##### Hover walk

In this task, participants walked on 4.5 cm wide by 2 m long beam fixed to the floor. Participants’ score was the distance traveled in 45 s or when they fall off the beam. The best of two trials was counted.

##### Rope skipping

Participants began skipping for 15 s forward bipedally. At a signal, they had to change to skipping backward. A recorder noted the number of achieved jumps from the best of two trials.

##### ‘Juggling’ while standing on a balance board

Participants stood with one foot on a balance board using their “free foot” for balance. In one hand, they held a handball with their palm facing upward. On top of the handball in their hand, participants had to bounce another handball for 15s. The “free hand” could be used for balancing movements. One trial was performed with the right foot/hand and the second performed with the left foot/hand. The total number of bounces from both trials was recorded as the participant’s score on this task.

##### Roll with ball catching

In this task, participants laid on a gym mat on their back holding a handball with both hands at their chest. The task required the participants to throw the ball upward, roll 360° to the side and catch the falling ball. After this the same procedure was performed moving to the other side. The number of caught balls in 20 s in the best of two trials was recorded.

##### Benchhopping

Participants jumped alternately with the right and left foot on a 10 cm small beam placed between their legs 30 cm above the floor. Simultaneously, they had to bounce a handball on the beam, with the hand from the same side as the foot that is on the beam. If the participant lost control of the ball, a second one was immediately provided. The score was the highest number of bounces in 20 s across two trials.

##### Precision throw

A normal handball goal was covered so that only the upper corners of the goal (50 cm × 70 cm) were open. Standing at 9 m distance from the goal, participants had 30 s to make as much goals as they could with the number of trails and the number of achieved goals recorded as the score. The best of two trails was counted.

##### 30 and 100 m sprints

Participants had one trial each to sprint 30 and 100 m.

##### Long jump from a standing position

Participants had three trials to achieve the greatest distance in a standing long jump.

##### Jump and reach

The distance between reachable height in normal standing position and reachable height jumping with both legs was measured. The best of three trials was counted.

##### Handball long throw

This task required participants to throw the handball as far as possible. They were allowed to take a short run-up and the longest of three trials was counted.

### Part 2: Prediction by National and Regional Coaches

#### Participants

For this part of the study, participants included the two national coaches who observed the try-outs and the five regional coaches from the teams participating. All had the highest German handball coaching license and several years of experience coaching and selecting girls of this age group. Therefore, the information they could use were the observations during these try-outs as well as the data from the motor tests.

#### Procedures

All coaches were asked to nominate the best 14 players for an all-star game at the end of the try-outs. The national coaches selected together while the regional coaches discussed their selections with their trainer team (normally a team of two or three persons). Therefore, we received five separate nominations from the regional coaches, while the two national coaches decided jointly.

### Part 3: Predictions by Laypersons and Novices

#### Participants

Participants in the final phase of the experiment included 12 novice and 12 advanced handball players. The novice group consisted of six male and six female participants with a mean age of 22.8 years, *SD* = 3.4. They had no handball playing experience and only occasional experience watching handball games. The advanced group consisted of six male and six female handball players with a mean age of 31.3 years, *SD* = 12.7. Their average handball experience was 20.3 years of playing, *SD* = 10.7, and 1.9 years of coaching, *SD* = 2.9.

#### Stimulus Material

Given the differences in knowledge between the coaching and other groups, we decided to limit the information given to the lesser skilled groups. This was done since it was not possible to replicate the level of information national and regional coaches would be able to access in making their decisions (e.g., personal experience with athletes). To this end, the stimulus materials were videos from the handball talent section camp from 2001. During the camp, different teams (*n* = 5) played against each other in normal handball formation (six on six) and from these games video footage of offensive play from one half game was selected. This selection resulted in the creation of five different video pools, with each pool containing the matches of one team against the other four teams.

#### Procedures

Prior to commencement of the experiment, participants received verbal and written instructions and completed a questionnaire detailing their age and level of experience in handball. Subsequently, participants watched the video material on a TV set (Sony Trinitron) and selected who they felt were the most talented players. When participants perceived a talented player, they said “stop” at which time shirt number and playing position of the selected person as well as the time of participants’ verbalization were written down by the experiment supervisor. The five different video pools were presented in counterbalanced order. The duration of the offensive scenes per match (e.g., team one against team three) varied between 6–8 min, but showed exactly 5 min of playing time per match (i.e., with stoppage time). The experiment took approximately 160 min to complete and there was a 10-min break after the completion of three video parts.

### Data Analysis

Of the possible 68 female athletes, complete data sets for 58 players were obtained. These data were used to compare varying prediction models and the best model-fit from the motor tests. In contrast to other prediction studies in sports, we were less interested in the correlations between those predictions (Serwe and Frings, 2006; Scheibehenne and Bröder, 2007), and more in the type-I- and type-II-errors as well as the correct predictions. Therefore, we calculated simple cross tabulations for the prediction models. We considered a type-I-error as the proportion of incorrect prediction of a low achiever as one of the top 14 players and a type-II-error when later high achievers were not identified as top players (cf. **Table 1**). Because of the exploratory nature of this study and because we had no clear hypotheses regarding comparisons between the different models, we restrained from presenting inferential results and compare instead the correct classifications as well as both types of error.

**Table 1:** Possible outcomes of forecasts from a practical point of view.

	Low achievers	High achievers
Forecast as non-talent	Correct classification	aaaagray!80Type-II-error
Forecast as talent	aaaagray!50Type-I-error	Correct classification

To determine the best model-fit for the motor tests, a logistic regression analysis was calculated. The logistic regression analyses allowed for prediction of group membership on the basis of a set of variables. For the logistic regression analyses, these group memberships can be dichotomous and the variable mix can consist of continuous, discrete and dichotomous variables, which makes it more robust for predictions. The probability criterion was set from 0.15 to 0.20 to ensure that all important variables were included in the model.

## Results

### A-Priori-Probability

For the a-priori-probability, which was used as the baseline comparison for other models, all players were forecasted as not-talented. Therefore, 75.9% were correctly forecasted as low-achievers, while 24.1% were predicted incorrectly since they were high-achievers (type II error).

### Part 1: Prediction by Motor Test

**FIGURE 1:**
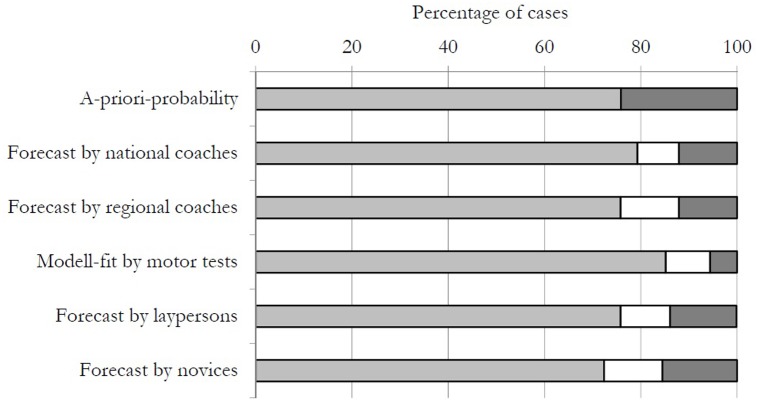
Comparison of varying forecasts in percent. The light grey bars indicate the correct predictions, the white ones the type-I-error and the black ones the type-II-errors.

Subsequently, a *post hoc* model fit was calculated on the basis of the motor test data (cf. **Figure 1**). As with previous studies, motor tests were considered a measure of general motor abilities and can therefore predict the motor potential for future performances (Schneider et al., 1993). Importantly, the statistical analysis conducted here was not prognostic *per se* (i.e., as the other models were) and should be kept in mind when interpreting these results. While this is an often-administered approach, testing the prognostic validity requires a two-step process. The first step requires generating the prediction formula of data from a first talent selection camp and then using this formula in a second step to determine its efficacy for predicting talent. However, in this study we only had data for the first step and therefore consider these results cautiously.

A forward stepwise logistic regression revealed “bench hopping” as a significant predictor in the first step with a correct classification of 74.1%, χ^2^(1) = 10.12, *p* < 0.01, *R*^2^ = 0.26. In the second “step roll with ball catching” was identified as a significant predictor and increased the correct classification to 79.6%, χ^2^(1) = 6.34, *p* = 0.01, *R*^2^ = 0.39. A third significant predictor, number of goals in the “precision throw task,” decreased the correct classification to 75.9%, χ^2^(1) = 2.49, *p* = 0.11, *R*^2^ = 0.44. Finally, number of throws in the precision throw task was identified in Step 4 and improved the correct classification to 85.2%, χ^2^(1) = 4.73, *p* = 0.03, *R*^2^ = 0.53. The number of correct classifications was higher than the a-priori-probability and the type-I-error (9.2%) was higher than the type-II-error (5.6%).

### Part 2: Prediction by National and Regional Coaches

Analysis of the national coaches indicated they correctly classified 79.3% of participants in the training camp, which is 3.4% more than the a-priori-probability. However, they had a type-I-error of 8.6% and a type-II-error of 12.1%. The regional coaches had a total of 75.8% correct classifications with 12.1% for both type-I- and type-II-errors.

### Part 3: Predictions by Handball Players and Novices

Next, the groups of handball players and novices were analyzed. In a first step, the optimal model fit was determined by testing varying numbers of nominations, similar to the method used with the regional coaches (cf. **Table 2**). For the players this was reached with five nominations and for the novices with six. The group of players were able to predict 75.8% correctly. Their type-II-error was 13.8% and the type-I-error was 10.3%. For the group of novices, the percentage of correct classifications went down to 72.4% and their type-II-error was 15.5% with type-I-error as 12.1%.

**Table 2:** Amount of nominations by varying predictors.

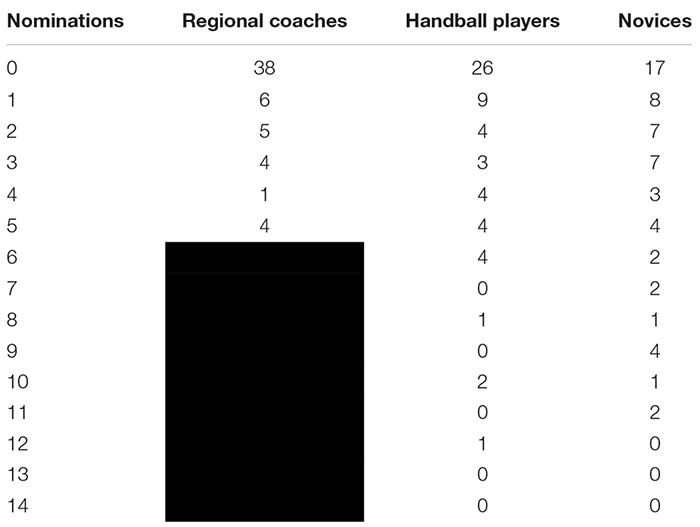

### Comparison of Predictions

Comparison of the different methods revealed that the best classification was by the *post hoc* model fit of the logistic regression of the motor test results (cf. **Figure 1**). The best forecast came from the national coaches, but they were only slightly better than the a-priori-probability. The advanced players performed with the same accuracy as the regional coaches and the forecast by novices was also quite similar. Comparing the type-II-error, the logistic regression on the motor tests outperformed all other models. Here, the regional and national coaches performed similarly but were only slightly better than the forecasts by the advanced players and laypersons.

## Discussion

This study compared talent selection and forecasting decisions across different levels of skill. Our results indicated only small differences in the classifications between the different models using varying predictors. As expected, the *post hoc* logistic regression using the motor tests outperformed all other forecast models, presumably because this approach used all available data to create the best model-fit. It needs to be restated here that it can only be considered cautiously, because it is no real prediction, but it provides us with an idea how much variance might be possible to explain. This caution increases even, when you see that the prognostic validity of the predictors seems questionable. From a practitioner’s viewpoint, these would likely not be the tests chosen as the most predictive of later performance because they do not measure handball-specific skills (e.g., handball distance throw). Future studies need to confirm the prognostic validity of these tests (cf. Lidor et al., 2009).

The slightly superior performance of the national coaches was interesting. On the one hand, national coaches should be significantly better at forecasting the future success of the talents they scout. However, on the other hand, they were at the try-out camp and used information collected over the 5 days to make their decision (as well as any personal experience they might have had); moreover, and perhaps more importantly, they may have had a role in influencing future performance by training the selected players in the national team and also identifying them as highly talented players for the professional clubs, which may have resulted in greater quantity and quality of training. The same holds for the regional coaches who are usually ‘active agents’ in the training of some of the athletes. In the light of these confounds, the issue is perhaps not that the well-educated and trained coaches were superior to the performance of the less educated/knowledgeable groups but that the differences between these groups were so small. Future studies are necessary to replicate these results, ideally with greater test power, and determine the influence confounding variables may have had.

In these future studies, some limitations should be overcome if possible. Most significantly, our design limited the information to which the advanced and novice groups had access. While this may have prevented lesser skilled decision-makers from being overwhelmed by the amount of data (see Goldstein and Gigerenzer, 2009), it resulted in a discrepancy in available knowledge between the groups for making their decisions. The coaches, for example, had access to information drawn from observations of the games played, results of the motor tests and through interactions with other coaches at the talent selection camp, not to mention any personal knowledge they may have had about the players. On the one hand, a qualitative study examining ‘how’ coaches make these decisions may be valuable. On the other hand, it may also be possible that these differences in information access could be avoided using more strict laboratory protocols (Marewski et al., 2010; Raab, 2012). It should be noted that while controlling information in lab settings might improve experimental control in the study design, it could decrease the ecological validity and representativeness of the tasks under examination. Collectively, the current results, coupled with those from future qualitative and experimental studies with more precise controls, may do much to explain the practice of talent prognosis.

Three points should be considered from a practical viewpoint. First, although our focus has been on comparing the different levels of assumed decision-making skill to a *post hoc* model, there may be some inherent value to the use of objective tests that was not explored in the present study. For instance, objective tests have a pedagogical aspect to them. By controlling for specific skills with these tests, every players gets feedback on his or her performance, something that can be very useful in the learning process (Shea and Wulf, 1999). The coach can tell each player where there is room for improvement and in which part of their performance they are already superior.

Second, it seems important to not only look at the percentage of correct classifications. Depending on your position in the sports system, type-I- or type-II-errors have different consequences. For instance, if the prediction model produces more type-I-errors, low achievers are supported and developed although they might not eventually reach elite levels of performance. However, in sport systems under financial constraints (i.e., most sport systems), these types of errors are not affordable. Prediction models that fail to identify an athlete with high potential result in lost talent, which can be particularly damaging if the numbers participating in the sport are low. While the effectiveness of talent development systems has been questioned (Vaeyens et al., 2009), the overall goal of athlete development systems is to facilitate the most talented persons to reach elite status; therefore, it is important to consider which type of error is more relevant for the improvement of the talent identification and development process.

Third, given the amount of error that is found in all predictions, it seems reasonable to create a system in which athletes are not only considered as potential high-performers at a single time point. Therefore, multiple testing or observations should be included in the talent system. For the German Handball federation, for example, this was implemented by having two national try-outs that are a year apart, as well as having the national coaches scout at elite level youth matches (Schorer et al., 2012). A similar point relates to the need for talent selection process to acknowledge the challenge in creating representative tasks that capture the nuanced relationships among skills that may be still developing (e.g., anticipation, decision-making) and performance outcomes (as a youth and as an adult) that may be predicted by different combinations of variables.

The use of longitudinal data for our analyses was a strength of this study. Most studies in sport consider how specific tests (e.g., anthropometric, cognitive or physiological) predict selection at one point in time. Little research has considered how early performance predicts achievement at later points of time (Güllich and Cobley, 2017), with still less considering how to forecast performance after 10 years of talent development, a time period noted as being an important threshold in the development of expertise (Ericsson, 2007; Ericsson and Williams, 2007; Ericsson et al., 2009). Despite this strength, there are some limitations to our analyses. First, the small number of participants resulted in goal keepers and field players being analyzed together despite the clear performance differences between these positions. Future studies should try to increase the number of observations and try to differentiate between playing positions. Additionally, the testing was conducted based on the standards from over 10 years ago and as a result some of the tests from 2001 are no longer considered as ‘state of the art.’

It is noteworthy that two athletes within the sample participating at the talent camp later became National team players. One of them, a goalie, was recognized by all of the coaches. The other, a field player, was not recognized by either of the national coaches and only two of the regional coaches. At the age under investigation here, she seemed to have nothing outstanding one could see. Anecdotally, she reported that she started training quite hard after the first selection camps and subsequently became a national team player. These examples illustrate how difficult the forecasting of future national players might be, because the process of talent development is often non-linear, highlighting again the problematic nature of type-II-errors (Renshaw et al., 2012).

In summary, talent selection is a very difficult task; ideally it is a decision that should be re-considered often, in order to consider performer-specific changes over development. Based on the results of the current study, understanding the foundation on which these decisions are, and should be, made seems an open field for future research in all kinds of talent domains.

## Ethics Statement

The study was conducted over 10 years ago. At this time the institute of the first author did not have an ethic committee. But the study was conducted in accordance with the ethical declaration of Helsinki. We received written informed consent from all participants that made decisions.

## Author Contributions

JS conceptualized and designed the study. He acquired, analyzed and interpreted the data. He also drafted substantial parts of the manuscript. RR and LF wrote and revised parts of the manuscript. JB conceptualized and designed the study. He also drafted substantial parts of the manuscript. All authors read and approved the final manuscript.

## Conflict of Interest Statement

The authors declare that the research was conducted in the absence of any commercial or financial relationships that could be construed as a potential conflict of interest. The reviewer AV and handling Editor declared their shared affiliation, and the handling Editor states that the process nevertheless met the standards of a fair and objective review.
